# The tandem–random transition of cellular patterning: proposed roles of N‐cadherin‐based orientational cell adhesions in the development, maintenance, and degeneration of the nucleus pulposus

**DOI:** 10.1111/brv.70081

**Published:** 2025-09-19

**Authors:** Xiangyun Wei, Nam Vo, Gwendolyn A. Sowa

**Affiliations:** ^1^ Department of Ophthalmology and Department of Microbiology & Molecular Genetics University of Pittsburgh 1622 Locust Street Pittsburgh PA 15219 USA; ^2^ Department of Orthopaedic Surgery, McGowan Institute for Regenerative Medicine, and Department of Pathology University of Pittsburgh 200 Lothrop Street Pittsburgh PA 15261 USA; ^3^ Department of Physical Medicine and Rehabilitation, Department of Orthopaedic Surgery, and Department of BioEngineering University of Pittsburgh 3471 5th Avenue Pittsburgh PA 15213 USA

**Keywords:** orientational cell adhesion, N‐cadherin, Cdh2, intervertebral discs, nucleus pulposus, notochord, low back pain, neck pain, Lego hypothesis of tissue morphogenesis

## Abstract

Intervertebral disc degeneration (IDD) can contribute to lower back and neck pain. In IDD, the most affected component of the intervertebral disc is the nucleus pulposus (NP). Derived from the notochord, where cells are organized into a tandem configuration, young NP cells cluster in three‐dimensional (3D) networks embedded in a gelatinous matrix. Here, we review the current understanding of NP development, homeostasis, physiology, and degeneration with a focus on the roles of the cell adhesion molecule N‐cadherin in these processes. Based on the literature, we hypothesize that N‐cadherin contributes to the architectural transition from the notochord to the NP by mediating a switch in cellular organization from tandem to random orientational cell adhesions (OCAs). We further hypothesize that the 3D clustering of NP cells may facilitate N‐cadherin to act as a mechanosensor to modulate NP gene expression under mechanical stresses. We hope these hypotheses promote future research on the etiology of human IDD and the development of measures to prevent and treat IDD. Some open questions on N‐cadherin functions in the NP are also discussed.

## I. INTRODUCTION

In humans, the intervertebral discs (IVDs) often undergo progressive degeneration, a condition that can start as early as the first few decades of life (Miller, Schmatz & Schultz, 1988). Intervertebral disc degeneration (IDD) is a common contributor to lower back and neck pain, and may affect up to two‐thirds of the population (Choi, Cohn & Harfe, 2008; Peng & DePalma, 2018; Raj, 2008). There is no cure and non‐operative treatments have variable outcomes; current surgical treatments aimed at targeting the sequelae of IDD are invasive and often unsatisfactory due to low efficacy and high risk of serious complications (Kos, Gradisnik & Velnar, 2019). As a result, the healthcare costs of lower back and neck pain in the USA are the highest among 154 health conditions, estimated at $134.5 billion in 2016 (Dieleman *et al*., 2020). This situation is difficult to change due to insufficient understanding of the basic biology of IVD development, homeostasis, and degeneration.

**Fig. 1:**
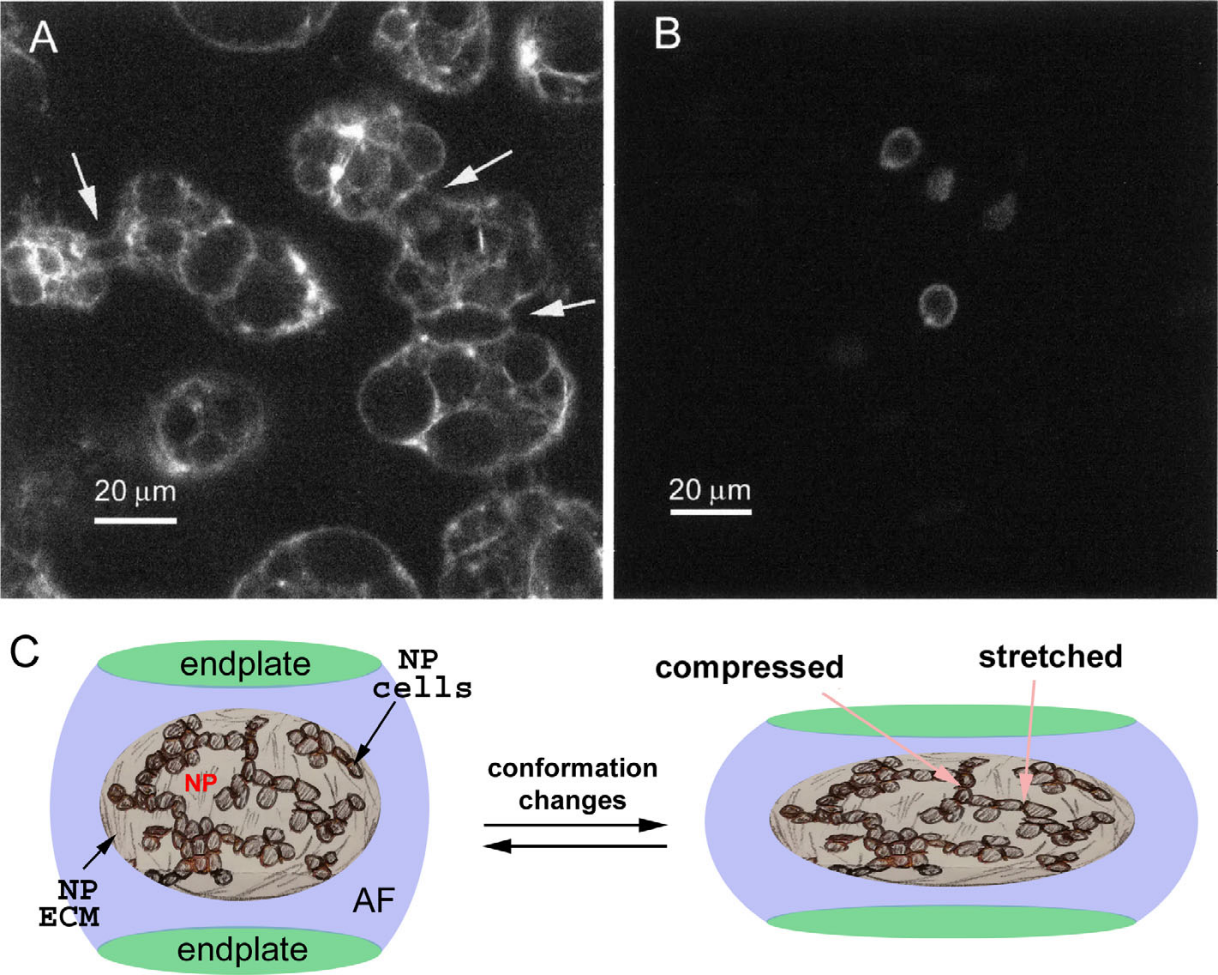
The components of the intervertebral disc and its deformation under mechanical stresses. (A) Phalloidin staining of Actin, illustrating that nucleus pulposus (NP) cells of a young non‐chondrodystrophoid dog (Thompson grade I disc) (Thompson *et al*., 1990) form interconnected clusters. Arrows indicate interconnectivity between larger cellular clusters. (B) In an aged non‐chondrodystrophoid dog (Thompson grade III disc), the dispersed and sparse NP cells suggest that the three‐dimensional (3D) cellular clustering is lost. (C) Illustration of the structure of the intervertebral disc, with the gelatinous NP surrounded by the cartilaginous annulus fibrosus (AF) and two endplates. Under mechanical stresses, the NP deforms, transferring tensile, compressive, shear, and/or torsional stresses to the NP cells, which are connected in 3D networks embedded in the extracellular matrix (ECM). Images in A and B are reproduced from Hunter *et al*. (2003a), with permission from *Tissue Engineering*.

Each IVD has four structural components: a central gelatinous nucleus pulposus (NP); a fibrocartilaginous annulus fibrosus (AF), which encircles the NP; and two cartilaginous endplates with one positioned cranial to and the other caudal to the AF and NP (Lawson & Harfe, 2015). In age‐related IDD, the NP is commonly affected. The NP acts as an elastic cushion to absorb mechanical impacts, thus playing a vital role in the functions of IVDs (Hunter, Matyas & Duncan, 2003a, 2004b; Lawson & Harfe, 2015; Palacio‐Mancheno *et al*., 2018; Raj, 2008; Trout, Buckwalter & Moore, 1982a). The critical physical properties of the NP are largely determined by the extracellular matrix (ECM) secreted by NP cells, which are embedded in the ECM and cluster in three‐dimensional (3D) networks in many mammalian species through intercellular cell–cell adhesions (Fig. 1); this 3D clustering is observed in young NP but not in aged NP, where NP cells exist mostly as isolated individual cells (Hunter *et al*., 2003a, 2004b; Lawson & Harfe, 2015; Palacio‐Mancheno *et al*., 2018; Raj, 2008; Trout *et al*., 1982a). The deformation resulting from mechanical load imposes mechanical strains on NP cells at their cell–cell intercellular adhesions or cell–matrix adhesions, which may be coupled to mechanotransduction to promote gene expression for NP maintenance (Fig. 1C). Thus, the organization of NP cells, their cell–cell, and cell–ECM adhesions all potentially play an important role in responses to mechanical stresses, and the changes in 3D clustering observed with aging may be associated with alterations to the NP matrix.

**Fig. 2:**
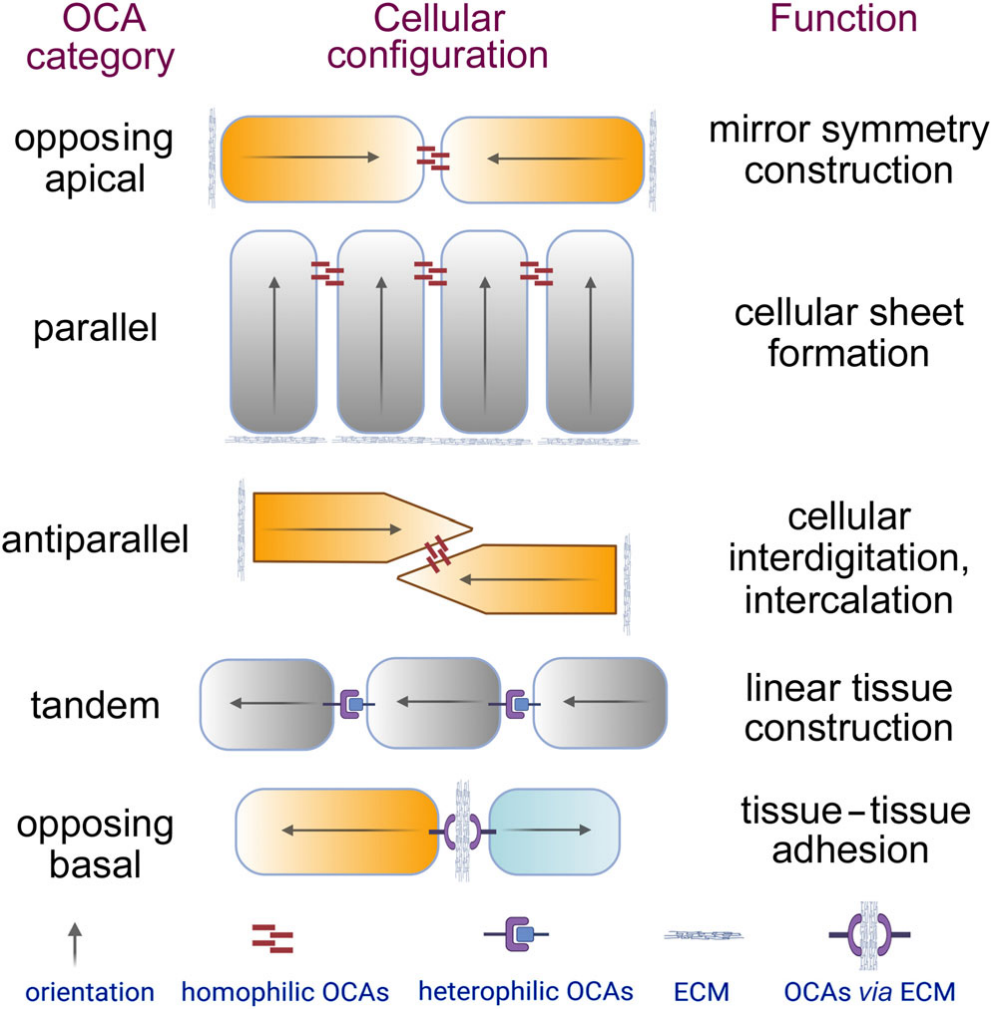
Orientational cell adhesions (OCAs), i.e. cell–cell and cell–matrix–cell adhesions that underlie various orientational relationships between the intrinsic polarities of the coalesced cells. They can be classified by the distinct orientational intercellular relationships they define. The diagrams illustrate five categories of OCAs that organize the basic configurations of cells that display apicobasal polarity and planar cell polarity. Cells are illustrated as boxes. Arrows and colour‐shading gradients represent the orientations of the inherent cell polarities. ECM, extracellular matrix (depicted here as the basement membrane). Adapted from Zhang & Wei (2022), with permission from *Trends in Cell Biology*.

The organization of NP cells depends on cell adhesions, which can be described and studied from the perspective of the concept of orientational cell adhesions (OCAs) and the Lego hypothesis of tissue morphogenesis. OCAs refer to cell–cell or cell–matrix–cell adhesions that define the relative orientations of the intrinsic polarities of neighbouring cells; such orientational intercellular relationships underlie various cellular configurations in tissues (Fig. 2) (Zhang & Wei, 2022, 2023, 2024). Evolving from the OCA concept, the Lego hypothesis suggests that the topographical properties of cell surface adhesion molecules can be dynamically altered and polarized by regulating the spatiotemporal expression and localization of OCA molecules cell‐autonomously or non‐cell‐autonomously through cell–cell interactions, thus modulating cells into unique ‘Lego pieces’ for self‐assembly into distinct tissue architectures (Zhang & Wei, 2025). By coupling cell adhesions with the orientation of inherent cell polarities, the recently proposed concepts of OCAs and the Lego hypothesis offer a new perspective to understanding tissue morphogenesis, maintenance, and degeneration, although the final shape, architecture, and maintenance of a tissue will also depend on other cell behaviours that influence the critical mass and physical properties of its constituent cells. These cell behaviours include but are not limited to cell proliferation, orientation of cell division, cell death, cell differentiation, cell–matrix and cell–cell interactions that modulate cell polarities, cell migration, and modulation of cell shapes and sizes (Green, 2022), and these cell behaviours are not isolated but rather they mutually influence each other. For example, cell–matrix interactions can alter the orientations of cell polarity and cell–cell adhesions (Thery & Bornens, 2006; Tseng *et al*., 2012), and local sensing of force or geometry can affect many cellular behaviours such as cell growth, differentiation, and cell death (Vogel & Sheetz, 2006).

OCAs can be categorized according to the types of orientational intercellular relationships they define. The OCA concept showcases the capability of an adhesion molecule to take on different roles in mediating various intercellular orientational relationships in different cellular contexts. Depending on the cellular and environmental contexts, the same adhesion molecule can act in different types of OCAs to promote the formation of various tissue architectures according to the Lego hypothesis of tissue morphogenesis. For example, in the established neural epithelium, the N‐cadherin cell adhesion molecule is concentrated at the adherens junctions and plays an essential role in aligning neighbouring cells in a parallel configuration for cellular sheet formation, whereas in the early neural rod, N‐cadherin can mediate opposing apical OCAs between the apices of contralateral cells to facilitate their opposing configuration for the formation of the mirror‐symmetric neural rod (Guo *et al*., 2018; Zhang & Wei, 2023). N‐cadherin, also called cadherin 2 (Cdh2), belongs to the type I subfamily of the cadherin superfamily (Nollet, Kools & van Roy, 2000). N‐cadherin mediates versatile cell–cell adhesion *via* homophilic adhesion between itself and other N‐cadherin molecules or heterophilic adhesion with other molecules at comparable adhesion strengths (Prakasam, Maruthamuthu & Leckband, 2006). Evidence for N‐cadherin expression in the notochord (Hatta *et al*., 1987; Hatta & Takeichi, 1986; Inuzuka, Redies & Takeichi, 1991; Lin, Wang & Redies, 2014; Radice *et al*., 1997; Sakamoto *et al*., 2008; Simonneau, Broders & Thiery, 1992; Warga & Kane, 2007) and NP (Chen *et al*., 2015; Hwang *et al*., 2016, 2015; Lv *et al*., 2014) suggests that N‐cadherin also plays important roles in the development and physiology of the NP as well as its precursor, the notochord. Due to architectural differences between the notochord and the NP, N‐cadherin may function in different types of OCAs.

The expression and function of N‐cadherin in the NP has been an active research topic with respect to IDD. From the perspective of the OCA concept, this review summarizes the current understanding of NP development and maintenance, focusing on two aspects. First, we review some general features regarding how the notochord may transform into the NP by adjusting OCAs and how NP cellular architecture may be involved in NP maintenance. Second, we consider how N‐cadherin may be involved in NP development and maintenance. We synthesize the existing literature and propose the hypothesis that N‐cadherin contributes to the architectural transition from the notochord to the NP by mediating a switch in cellular organization from tandem to random OCAs. A better understanding of N‐cadherin functions in NP physiology has the potential to help with developing effective prevention and treatment strategies for lower back and neck pain caused by IDD by identifying novel treatment targets.

## II. SOME GENERAL FEATURES OF NP DEVELOPMENT AND MAINTENANCE

### (1) Transition from the notochord to the nucleus pulposus

The vertebrate NP is derived from a morphologically distinct tissue: the notochord. The notochord is the defining midline axial organ of the phylum Chordata, which traditionally includes three subphyla: Tunicata or Urochordata, Cephalochordata, and Vertebrata (Satoh, Rokhsar & Nishikawa, 2014). The craniocaudally elongated, rigid, and yet flexible notochord is composed of cells arranged in tandem as a rod‐shaped tissue; it supports embryonic structural integrity and movement (Corallo, Trapani & Bonaldo, 2015; Stemple, 2005). In addition, the notochord is an organizer that secretes signals to orchestrate the development of many surrounding tissues (Placzek, 1995; Saude *et al*., 2000).

**Fig. 3:**
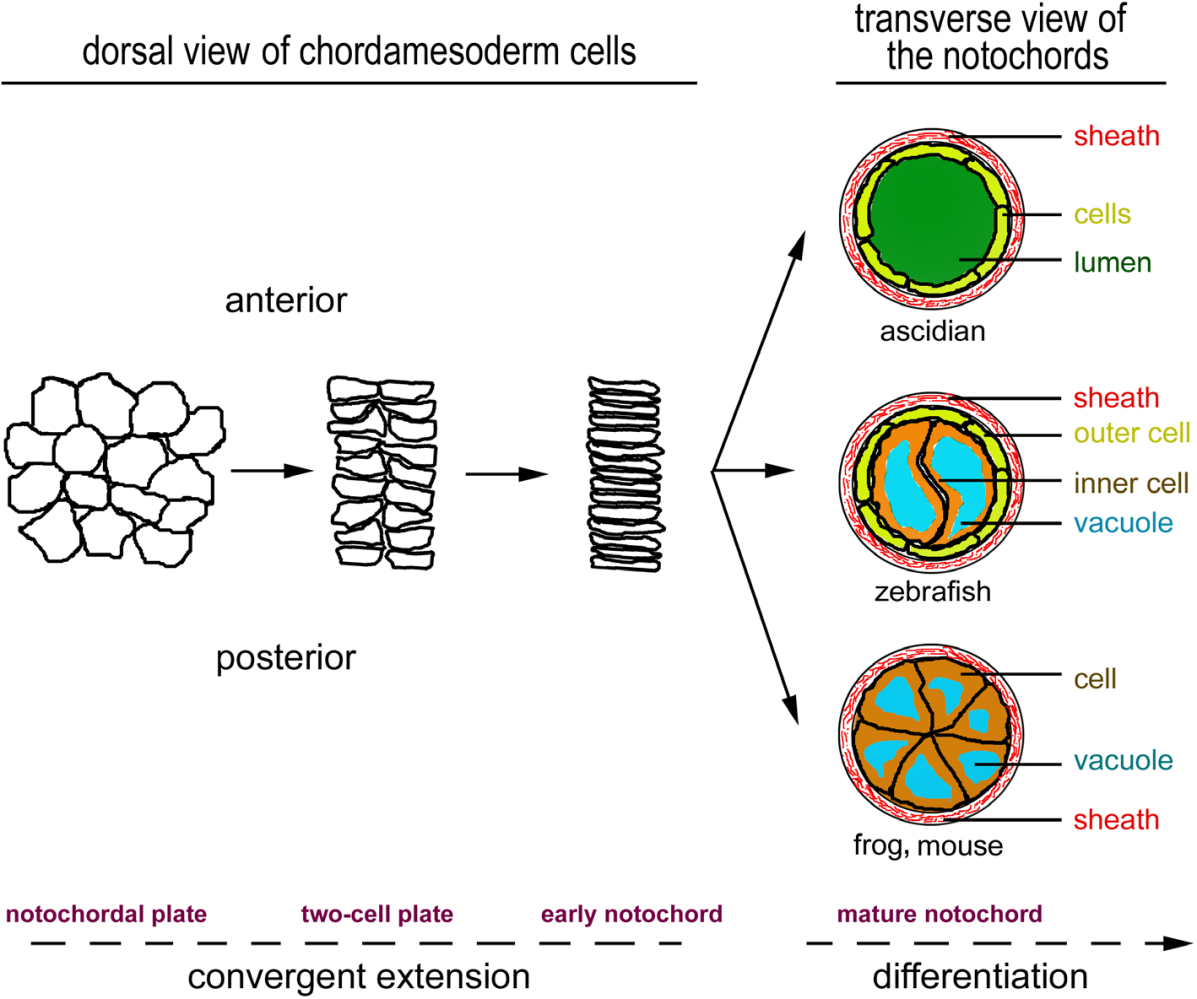
Three modes of notochordal development in chordates. The convergent extension phase of notochordal development rearranges the chordamesoderm cells of the notochordal plate into the immature notochord consisting of a file of cells of single‐cell width. This phase can be divided into three stages: the notochordal plate, the two‐cell notochordal plate, and the immature notochord. In the differentiation phase, the immature notochord develops by three different modes in different species into distinct types of extracellular matrix (ECM) sheath‐enclosed mature notochord: the hollow notochord in ascidians, the centre‐surround patterned rod‐shaped notochord in teleost fish, and a solid rod‐shaped notochord composed of wedge cells in amphibians and mammals.

Notochordal development can be divided into four stages according to the cytoarchitectural features unfolding during morphogenesis: (*i*) the notochordal plate; (*ii*) the two‐cell‐wide notochordal plate; (*iii*) the immature notochord consisting of a file of cells of single‐cell width; and (*iv*) the mature notochord which is enclosed within an ECM sheath. These four stages can be organized into two phases: a convergent extension phase followed by a differentiation phase (Fig. 3). During the convergent extension phase, the wide notochordal plate reorganizes to form the immature early notochord *via* mediolateral cell intercalation. This process is governed by many apicobasal and planar cell polarity proteins (Balmer, Nowotschin & Hadjantonakis, 2016; Heisenberg & Tada, 2002; Jiang & Smith, 2007; Keller *et al*., 2000; Kida *et al*., 2007; Kourakis *et al*., 2014; Munro, Robin & Lemaire, 2006; Munro & Odell, 2002; Ninomiya, Elinson & Winklbauer, 2004; Shindo, 2018; Wallingford, Fraser & Harland, 2002; Zallen, 2007). During the subsequent differentiation phase, immature notochordal cells differentiate and reorganize into the mature notochord *via* three modes that differ among chordates (Fig. 3). (*i*) In ascidians, notochordal cells reshape and reorganize to form a hollow tube, which contains an extracellular lumen in which osmotic pressure maintains mechanical rigidity (Dong *et al*., 2009; Jiang & Smith, 2007; Munro *et al*., 2006). (*ii*) In frogs, rodents, and humans, notochordal cells take on a wedge shape in transverse section to assemble into a solid rod‐shaped tissue (Balmer *et al*., 2016; de Bree, de Bakker & Oostra, 2018; Keller *et al*., 2000; Murakami *et al*., 1985; Shinohara & Tanaka, 1988). (*iii*) In zebrafish (*Danio rerio*), notochordal cells differentiate into two types of cells, organized in a centre‐surround configuration, with the inner vacuolated cells surrounded by non‐vacuolated endothelial‐like outer sheath cells (Glickman *et al*., 2003; Norman *et al*., 2018; Stemple, 2005). In all three modes, the outcome of notochordal morphogenesis is the production of a rod‐shaped tissue architecture, with its constituent cells organizing largely in a tandem configuration along the craniocaudal axis. The nature of the tandem OCAs that direct the assembly of the rod‐shaped notochord is yet to be elucidated. However, some evidence from ascidians suggests that planar cell polarity adhesion proteins are involved, likely in collaboration with other non‐traditional planar cell polarity adhesion proteins. For example, in *Ciona savignyi*, the planar polarity proteins Prickle and Dishevelled localize to the anterior edges of the notochordal cells. This polarized distribution suggests that they participate in mediating adhesion to the posterior edges of preceding anterior cells, thus generating the tandem cellular configuration of the notochord (Jiang, Munro & Smith, 2005).

The rod‐shaped embryonic notochord is short‐lived in vertebrates; in adults, the notochord regresses and transforms from a single tissue entity into the segregated NPs or a constricted but connected NP strand, depending on species. This transformation was demonstrated by two landmark cell lineage‐tracing studies in mice (Choi *et al*., 2008; McCann *et al*., 2012). In young NP, cells are no longer aligned in tandem; rather, they cluster into 3D cellular networks, suggesting extensive physical coupling among NP cells (Fig. 1A) (Hunter *et al*., 2003a, 2004b; Lawson & Harfe, 2015; Palacio‐Mancheno *et al*., 2018; Raj, 2008; Trout *et al*., 1982a). This architectural transition from the tandem rod shape of the notochord to the random 3D cellular clusters of the NP occurs after the body plan has been laid out and major organs have formed in late embryogenesis. For example, in mice, at embryonic day 12.5 (E12.5), the segmentation of the tandem notochord has already occurred and it is fully segmented by E16.5, suggesting that these drastic cellular configuration changes occur after organogenesis (Choi *et al*., 2008; McCann *et al*., 2012). This timeline makes sense because the function of the notochord as the initial ‘skeleton’ to sustain embryonic integrity is fully replaced by the skeleton in the late stages of embryogenesis. Like notochordal cells, young NP cells have vacuoles. In addition to the formation of the NP, the notochord also contributes to other aspects of spine development. Recent studies in zebrafish showed that the notochord sheath cells are segmented along the craniocaudal axis into alternating domains: the cartilaginous domains, which align with the future IVDs, and the mineralizing domains, which recruit osteoblasts to form the vertebral bodies (Wopat *et al*., 2018).

**Fig. 4:**
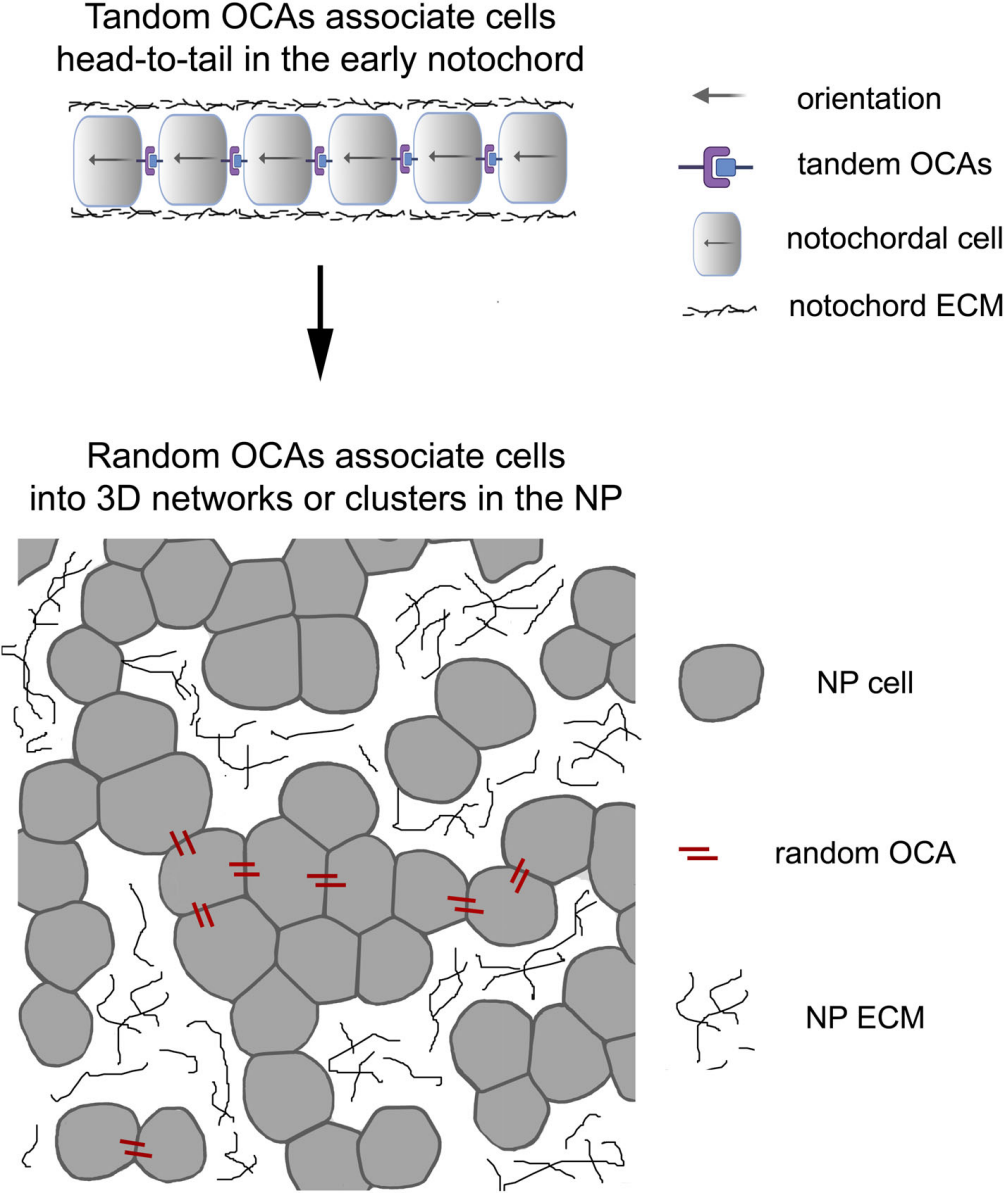
Based on the Lego hypothesis of tissue morphogenesis (Zhang & Wei, 2025), the replacement of tandem orientational cell adhesions (OCAs) with random OCAs is hypothesized to be responsible for the architectural transition from the linear configuration of the early notochordal cells to the three‐dimensional (3D) cellular clustering of the nucleus pulposus (NP) cells. To simplify the diagrams, not all random OCAs between NP cells are illustrated. The orientation of the intrinsic polarities of NP cells is not indicated because little is known about how NP cells are polarized and how random OCAs are distributed relative to the intrinsic polarities of NP cells. The random OCAs are expected to be impacted by mechanical stresses when cells are stretched, compressed, sheared, or twisted. ECM, extracellular matrix.

Despite the above findings, one important question remains: how does the tandem and continuous notochordal cellular configuration transform into the segregated and randomly clustered 3D networks in the NP? Considering the architectural transition, we propose that in the early notochord, a set of tandem OCAs are responsible for aligning notochordal cells in a head‐to‐tail configuration, in which the intrinsic polarities of individual notochordal cells are aligned in the same direction along the craniocaudal axis. By contrast, in young NP, the orientations of the intrinsic polarities of NP cells are likely disorganized, with OCAs distributed in a random fashion. As a result, NP cells aggregate into 3D clusters, either appearing as networks of interconnected NP cells as observed in mammals (Hunter *et al*., 2003a) or large masses of cell aggregations in teleost fish (Butylina *et al*., 2023; Haga, Dominique & Du, 2009; Irie *et al*., 2016; Schmitz, 1995) (Fig. 4).

### (2) Cellular organization in aged and degenerated NP

The transformation in cytoarchitecture from the notochord to the NP may be important for NP functions because cellular clustering is lost during IVD aging and/or degeneration (Hunter *et al*., 2003a, 2004b; Hunter, Matyas & Duncan, 2003b, 2004a). Although in young vertebrates, NP cells form 3D network clusters, which are embedded in the ECM composed largely of water, collagen II, and proteoglycan aggrecan (Hunter *et al*., 2003a), NP cells are more dispersed and resemble chondrocytes morphologically in aged animals, where they are small and do not contain the vacuoles observed in young NP cells (Hunter *et al*., 2003b, 2004a; Sakai *et al*., 2009) (Fig. 1B). These changes in cell morphology have been observed in many vertebrate species, including dogs, cats, rats, sheep, rabbits, pigs, ferrets, and mice (Hunter *et al*., 2004a), suggesting that it is a common phenotype of NP aging. Although NP cells have lost their original 3D clustering architecture in aged NP, some long subcellular tubular processes have been observed to connect these cells (Errington *et al*., 1998). These processes may allow a degree of intercellular communication between NP cells, but the level of mechanical coupling *via* intercellular adhesion is unlikely comparable to that of 3D cellular clustering in young and healthy NP. In addition to cell dispersion, cellularity is also reduced in degenerated NP (Hunter *et al*., 2003a; Trout *et al*., 1982b), further disrupting the 3D clustering architecture.

### (3) A hypothesis for the function of NP cell clustering

The 3D clustering of NP cells (Hunter *et al*., 2003a,b, 2004a,b; Sakai *et al*., 2009) is unique to the NP and is not observed in the AF and endplates. The function of this 3D cellular organization of the NP is unknown. However, it is tempting to speculate that it enables NP cells to sense, respond to, and tolerate mechanical stresses. Under mechanical loads and multidirectional movements of the spine, the NP deforms in 3D, passing the mechanical stresses to NP cells. Thus, if these cells are responsible for sensing mechanical stresses during normal physiology, they may require mechanisms based on the stretching, compression, shearing, or torsion of NP cells (Fig. 1C). The 3D clustering of NP cells organized by random OCAs could therefore ensure that there are always some NP cells connected and aligned in the suitable orientation to sense a particular type of stress. However, we do not rule out potential mechanosensation mediated by interactions between NP cells and the ECM as cell–matrix adhesions are known to be involved in mechanosensing (Gauthier, Masters & Sheetz, 2012).

**Fig. 5:**
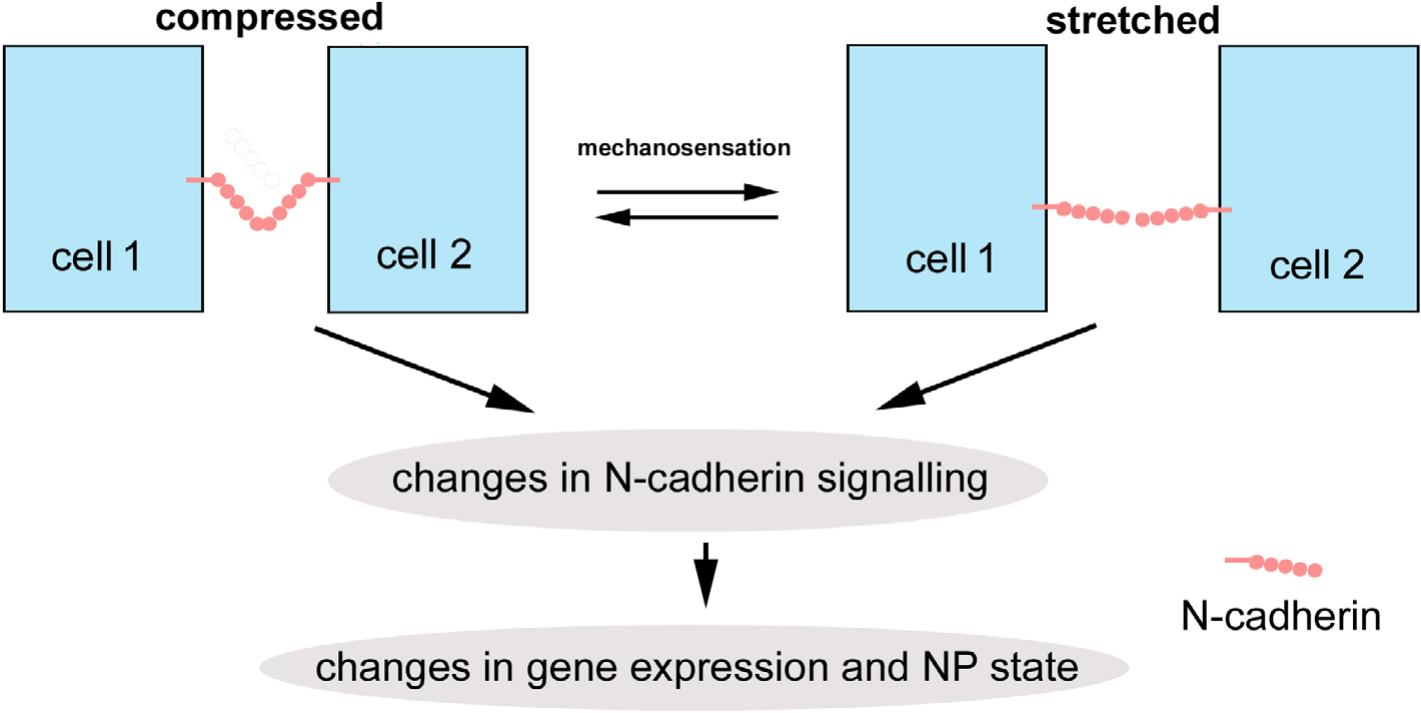
A model to explain the potential function of N‐cadherin as a mechanosensor in the nucleus pulposus (NP). N‐cadherin‐based potential random orientational cell adhesions (OCAs) sense mechanical stresses through conformational changes to elicit mechanotransduction to change gene expression and NP properties to cope with mechanical stresses. Mechanical stresses may be either beneficial or detrimental to NP health, depending on the degree of stress.

Thus, we hypothesize that NP cells sense mechanical stresses by monitoring conformational changes of intercellular adhesions, which can be dynamically affected by tensile, compressive, shear, and torsional mechanical stresses. This mechanosensation leads to changes in gene expression through mechanotransduction (Sowa *et al*., 2011) to adjust NP physical properties in response to mechanical stresses (Fig. 5). Such stress‐sensing capability is likely compromised or lost in aged and degenerated IVDs because the NP cells are more dispersed (Gruber & Hanley, 2002; Hunter *et al*., 2003a, 2004a,b; Trout *et al*., 1982b) and lack the intercellular adhesions required for mechanosensation. This reduction in stress‐sensing capability may make NP cells unable to adjust their gene expression under stress, compromising the maintenance of NP cells and the ECM. This chain of events could eventually lead to the failure of the NP.

According to the Yin‐Yang philosophy, mechanical stresses may be either beneficial or detrimental to NP health, depending on the interactions between stresses and NP cells. For example, high mechanical loads can cause a reduction in the expression of NP cell genes, including N‐cadherin, aggrecan, collagen II, Brachyury, laminin, keratin 19, glypican‐3 (Sowa *et al*., 2011). Thus, knowledge about how NP cells sense and respond to stresses at the molecular level may be central to understanding IVD health and degeneration.

## III. N‐CADHERIN IN NP DEVELOPMENT AND MAINTENANCE

### (1) N‐cadherin in notochordal development

N‐cadherin is expressed in the notochord in all vertebrate species examined to date by *in situ* hybridization and/or immunostaining, including frogs (Simonneau *et al*., 1992), zebrafish (Warga & Kane, 2007), chickens (Hatta *et al*., 1987; Inuzuka *et al*., 1991; Lin *et al*., 2014), rats (Hatta & Takeichi, 1986; Sakamoto *et al*., 2008), and mice (Radice *et al*., 1997), suggesting that it has conserved functions in the vertebrate notochord. Loss of N‐cadherin results in deformation of the notochord (Warga & Kane, 2007); this suggests that N‐cadherin is required for notochordal cells to organize into the correct rod‐shaped architecture, possibly by mediating tandem OCAs. However, evidence for this possible function is not yet available because the membrane localization of N‐cadherin makes it challenging to determine which of the cohered cells expresses N‐cadherin and whether it mediates homophilic or heterophilic adhesion (Prakasam *et al*., 2006). To verify this OCA function of N‐cadherin, its localization needs to be examined at both cellular and subcellular levels.

The involvement of N‐cadherin in notochord formation may also depend on interactions between the notochord and the surrounding tissues (Placzek, 1995; Saude *et al*., 2000). For example, the neural tube and the notochord are known to influence each other's development (Corallo *et al*., 2015; Stemple, 2005), and N‐cadherin is expressed in the neural tissue and is required for neurulation (Guo *et al*., 2018; Harrington *et al*., 2007; Hong & Brewster, 2006; Malicki, Jo & Pujic, 2003; Radice *et al*., 1997; Warga & Kane, 2007). In addition, the transition from the notochord to the NP depends on the surrounding sclerotomes, which are expected to provide initial segmentation signals to the notochord. Conversely, the notochord is required for the sclerotomes to re‐segment to form the vertebrae and the AF (Ward *et al*., 2018). However, this reciprocal dependence may not require direct N‐cadherin‐mediated cross‐talk between the notochord and the sclerotomes. N‐cadherin is downregulated during the development of the sclerotome (Duband *et al*., 1987) and AF tissue (Minogue *et al*., 2010; Risbud *et al*., 2015; Sakai *et al*., 2009), even though N‐cadherin is required for the initial formation of the somite, of which the sclerotome is a part (Duband *et al*., 1987). Thus, to what extent N‐cadherin regulates notochordal development in a tissue‐autonomous or tissue‐non‐autonomous fashion remains to be determined.

Regardless, the defects in tandem cellular configuration of the notochord upon the loss of N‐cadherin are consistent with our hypothesis that N‐cadherin may be involved in formation of the tandem OCAs that are responsible for adhering notochordal cells in a head‐to‐tail orientation along the anterior–posterior axis, either by directly contributing to tandem OCAs or by indirectly regulating them *via* the desmosomes observed between notochordal cells (Tong *et al*., 2013). Testing this hypothesis will require high‐resolution imaging of N‐cadherin localization during notochordal development.

### (2) N‐cadherin expression in the NP

The transition from the notochord to the NP does not eliminate the expression of N‐cadherin. N‐cadherin has been identified in the NP of many vertebrate species, including humans (Zhang *et al*., 2020), cows (Minogue *et al*., 2010), pigs (Hwang *et al*., 2016), rats (Tang, Jing & Chen, 2012), and mice (Zhang *et al*., 2019). This conservation of expression suggests that N‐cadherin plays an essential role in the NP. In fact, besides keratin 19, N‐cadherin is uniquely expressed in NP cells but not in AF cells and articular chondrocytes in many vertebrate species, even though these three cell types express other genes in common (Minogue *et al*., 2010; Risbud *et al*., 2015; Sakai *et al*., 2009). These expression characteristics suggest that N‐cadherin is a unique marker for NP cells in IVDs. It is not yet clear how N‐cadherin is involved in the mechanical load function of NPs at the tissue level, which is mediated by its ECM. The IVD endplates and AF can respond to the same mechanical loads and stresses experienced by the NP by using N‐cadherin‐negative chondrocytes and fibroblasts to maintain their ECM (Sakai *et al*., 2009), whose proteoglycan to collagen ratio (measured as the ratio of glycosaminoglycans to hydroxyproline) is about 2:1 compared to the much higher 27:1 for NP ECM (Mwale, Roughley & Antoniou, 2004). The conserved expression of N‐cadherin in the NP implies that N‐cadherin mediates unique NP functions that cannot be supported by N‐cadherin‐negative cells.

### (3) Roles of N‐cadherin in the 3D clustering of NP cells

Given that N‐cadherin can mediate cell adhesions, we hypothesize that it may contribute to random OCAs by localizing to NP cell membranes randomly, thus promoting the 3D clustering of NP cells. This hypothesis is consistent with observations that suppression of N‐cadherin by either antibodies or CRISPR (clustered regularly interspaced short palindromic repeats) mutations inhibits the formation of NP cell clusters in cell‐ and tissue‐culture analyses (Hwang *et al*., 2016, 2015; Niu *et al*., 2018; Palacio‐Mancheno *et al*., 2018). Furthermore, in aged NP where expression of N‐cadherin is significantly lower than that of juvenile NP, the NP cells become dispersed (Hunter *et al*., 2003a,b, 2004b; Hwang *et al*., 2016, 2015). Nevertheless, this hypothetical function of N‐cadherin in the NP has not been verified by *in vivo* approaches to date. Because the 3D clustering of NP cells is drastically different from the tandem configuration of notochordal cells, it is not unreasonable to hypothesize that N‐cadherin contributes to the architectural transition from the notochord to the NP by switching from mediating tandem OCAs to random OCAs in NP cells, whose intrinsic polarity orientations are not identifiable or may be aligned randomly relative to each other. This functional switch may depend on changes in the subcellular localization of N‐cadherin relative to the intrinsic polarities of NP cells.

The interconnections of NP cells are expected to involve desmosome adhesions, which display characteristic ‘spotty’ structural features under electron microscopy. However, unlike their strict distribution to lateral cell membranes in epithelia, the desmosomes in NP cells appear to be widely distributed (Schmitz, 1995; Trout *et al*., 1982b), suggesting they are randomly localized on the cell membranes. This distribution pattern qualifies desmosomes in the NP as a type of random OCA that may underlie the 3D network clustering of NP cells. Besides desmosomes, gap junctions have also been identified between NP cells (Hunter *et al*., 2004b). It is unclear whether N‐cadherin is part of the NP desmosomes, required for the formation or distribution of NP desmosomes and gap junctions, or independently mediates a distinct type of random OCA. Nevertheless, given the observation of N‐cadherin‐based spotty opposing apical OCAs at the apical surfaces of neural rod cells (Guo *et al*., 2018), it would not be surprising if N‐cadherin mediates NP cell assembly by directly forming spotty random OCAs, which could resemble the desmosomes observed under transmission electron microscopy (TEM). Verification of this proposed function will require mapping of the distribution of N‐cadherin on NP cell membranes at high resolution. We propose that testing the hypothesis that N‐cadherin mediates random OCAs in the NP, directly or indirectly, will provide important insights into the development, maintenance, and degeneration of the NP.

### (4) N‐cadherin as a mechanosensor in the NP

Besides cell–cell adhesion, any other biological functions of N‐cadherin in the 3D NP cell networks remain unclear. We argue above that 3D cellular clustering may underlie mechanosensation. Given that N‐cadherin may mediate random OCAs for 3D cellular clustering, we hypothesize that N‐cadherin in the random OCAs contributes to mechanosensation in the NP (Fig. 5), as it does in other biological contexts (Arslan *et al*., 2021; Baek *et al*., 2022; Inman & Smutny, 2021; Sheppard *et al*., 2023; Tortorella *et al*., 2022; Yap, Duszyc & Viasnoff, 2018; Zhang *et al*., 2021; Zhang *et al*., 2023; Zhang *et al*., 2022). This mechanosensation may be coupled to mechanotransduction *via* N‐cadherin‐mediated signalling (Yulis, Kusters & Nusrat, 2018). Although it is unclear what signalling pathways are involved, evidence suggests that β‐catenin‐regulated signalling (Hwang *et al*., 2016) and PI3K/Akt‐GSK‐3β (phosphoinositide 3‐kinase/protein kinase B‐glycogen synthase kinase‐3β) signalling (Li *et al*., 2017) may be involved. The challenge to verify whether these pathways are employed *in vivo* is the lack of animal models (Peng *et al*., 2006). Gene expression regulation controlled by the hypothesized N‐cadherin‐based mechanosensation and mechanotransduction may be the molecular basis for responses to mechanical stress. Furthermore, it may also underlie the differential gene expression between NP cells and chondrocytes that results in the NP being much more hydrated and less fibrous than the AF to buffer mechanical stresses.

### (5) N‐cadherin in NP degeneration

Evidence suggests that the expression levels of N‐cadherin are correlated with NP health status. During aging, NP gene expression profiles change (Chen, Yan & Setton, 2006). N‐cadherin and some other genes, such as *SNAP25*, *KRT8*, and *KRT18*, are expressed at higher levels in young and healthy NP cells (which are large and vacuolated) than in aged NP cells (small and non‐vacuolated) (Chen *et al*., 2015; Hwang *et al*., 2016; Lv *et al*., 2014; Minogue *et al*., 2010; Tang *et al*., 2012). This correlation suggests that N‐cadherin may play an essential function in NP maintenance and homeostasis, particularly under mechanical stresses. Loss of N‐cadherin may reduce or eliminate the intercellular adhesions between NP cells and thus the ability to sense mechanical stresses. This may render NP cells unable to adjust their gene expression to modulate cell organization and ECM properties to cope with such stresses. Supporting this, loss of N‐cadherin reduces the expression of aggrecan, collagen II, and Brachyury in cell‐culture conditions (Hwang *et al*., 2016, 2015). Needle puncture injury to mouse tail IVDs can also lead to reduced expression of N‐cadherin in the NP and cause NP degeneration (Zhang *et al*., 2019). However, this injury model may not represent the natural condition of age‐associated degeneration. Moreover, N‐cadherin was reported to inhibit the nuclear translocation of the yes‐associated protein (YAP) transcription factor to activate gene expression; downregulation of N‐cadherin led to stiff‐matrix‐induced NP cell death during NP fibrosis through ferroptosis (Ke *et al*., 2023). By contrast, overexpression of N‐cadherin *in vitro* promotes the resilience of NP cells to senescence caused by compressive stresses, as manifested by increased cell proliferation, reduced expression of senescence markers p16 and p53, increased telomerase activity, and upregulation of the matrix proteins collagen II and aggrecan (Hou *et al*., 2018; Niu *et al*., 2018; Wang *et al*., 2017). Taken together, the negative correlation between N‐cadherin expression levels and NP degeneration suggests that altering N‐cadherin expression levels may represent a novel treatment target for NP degeneration.

## IV. CONCLUSIONS

(1) We summarize the current understanding of the general features of NP development and maintenance and the roles of N‐cadherin in these processes. We propose the following hypotheses for future investigation.
(2) Tandem OCAs may be responsible for the rod‐shaped notochord, whereas random OCAs underlie the 3D clustering of NP cells. A particular cell adhesion molecule may function either uniquely in one type of these OCAs or differentially in both OCA types.
(3) In the notochord, N‐cadherin may contribute to the tandem organization of notochordal cells into a rod‐shaped tissue to support embryonic development and locomotion by acting as a tandem OCA molecule, whereas in the NP, N‐cadherin may be required for the 3D cluster network of NP cells by acting as a random OCA molecule. Addressing the question of how N‐cadherin transitions between these two roles may help to understand better the development and maintenance of the NP.
(4) N‐cadherin may also act as a mechanosensor to actively regulate NP gene expression to accommodate mechanical stresses. Addressing how this mechanotransduction is accomplished may offer important insights into NP maintenance and degeneration.
(5) Strategies to manipulate N‐cadherin expression may offer ways to treat lower back and neck pain associated with IDD.

## AUTHOR CONTRIBUTIONS

X.W. wrote the first draft of the manuscript. N. V. and G. A. S. edited and commented on the manuscript and suggested figure additions and revisions. X. W. drew and organized the figures and revised the final version of the manuscript.
